# Determinants of Risk Factors for Renal Impairment among HIV-Infected Patients Treated with Tenofovir Disoproxil Fumarate-Based Antiretroviral Regimen in Southern Vietnam

**DOI:** 10.1155/2020/7650104

**Published:** 2020-01-10

**Authors:** Cuong Q Hoang, Hai D Nguyen, Huy Q Vu, Khai T Nguyen, Linh T Hoang, Hong Ly, Thang D Tat, Lan T Phan

**Affiliations:** ^1^Ho Chi Minh City Pasteur Institute, Ho Chi Minh, Vietnam; ^2^Planning Department, Ho Chi Minh City Pasteur Institute, Ho Chi Minh, Vietnam; ^3^Medical Testing Centers, HCMC Medical University, Ho Chi Minh, Vietnam; ^4^Soc Trang HIV/AIDS Prevention Centers, Soc Trang, Vietnam; ^5^Medical Testing and Calibration Centers, Ho Chi Minh City Pasteur Institute, Ho Chi Minh, Vietnam; ^6^Ho Chi Minh City Pasteur Institute, Ho Chi Minh, Vietnam

## Abstract

**Background:**

The situation of renal impairment among HIV-infected patients treated with TDF-based antiretroviral (ARV) regimen greater than 3 years is little known when TDF use has been promptly increasing in Vietnam.

**Methods:**

We analyse demographic and clinical data from a cross-sectional survey of 400 HIV-infected patients aged ≥18 years, who were treatment-naive or switched TDF regimen within over 3 years between November 2018 and March 2019. Serological tests for serum creatinine, ALT, and AST were performed. Renal impairment was defined as an estimated glomerular filtration rate (eGFR) <60 mL/min/1.73 m^2^. Multivariate regression analyses were used to explore the risk factors associated with renal impairment.

**Results:**

At the baseline, 7.8% of respondents had estimated glomerular filtration rate (eGFR) of 30–59 mL/min/1.73 m^2^ and 0.8% had eGFR of 15–29 mL/min/1.73 m^2^, out of 34 (8.5%) of participants who had renal impairment. Multivariate analysis showed that participants who had preexposure to isoniazid (adjusted PR [aPR] = 0.35 Cl: 0.14–0.91) compared with nonexposure to isoniazid who had a BMI from 18.5 up to 25 kg/m^2^ (aPR = 0.31 Cl: 0.15–0.62) compared with BMI below 18.5 kg/m^2^ were less likely to suffer from renal impairment. Patients aged greater than 60 years (aPR = 26.75, 95% Cl: 3.38–211.62) compared with those aged 20–29 years were more likely to have increased risk of renal impairment.

**Conclusion:**

Our findings underscore the need for longitudinal studies to assess the influence of TDF on maintaining the low prevalence of renal impairment among HIV-infected patients in Vietnam.

## 1. Introduction

From the first HIV case detected in 1990 to June 30, 2016, there were 227,225 reported cases of HIV infection in Vietnam, of which the number of patients turned to AIDS was 85,753, and 89,210 HIV/AIDS infected cases died [1]. Since 2010, the World Health Organization (WHO) recommended tenofovir disoproxil fumarate (TDF) containing regimens for the treatment of HIV-infected patients; it was then used widespread and had officially become the preferred regimen [2] in Vietnam, since 2011, under the Decision 4139/QD-BYT of the Ministry of Health on amending and supplementing the guidelines for HIV/AIDS diagnosis and treatment. Accordingly, TDF, Lamivudine, and Efavirenz (TDF + 3TC + EFV) are the preferred treatment regimens for HIV/AIDS patients starting antiretroviral therapy (ARV), and now ARV regimens containing TDF are considered a top priority [3, 4]. Recently, Tenofovir alafenamide (TAF) has been accepted for use in several countries [5], although it has been proved to have an improved renal safety compared to TDF, if made available, in rural limited resources settings where regular renal function monitoring may be unrealistic [5]. According to Decision No. 5418/QD-BYT dated December 1, 2017 [3] of the Ministry of Health, Vietnam on issuing guidance for HIV/AIDs treatment and care, TAF has not yet been used for regular treatment in the health care centers in Vietnam. Besides, Abacavir was included in the treatment regime; it is often only assigned among HIV/AIDs children, coinfected with tuberculosis. Among adult HIV/AIDs patients, therefore, TDF is still quite popular in Vietnam.

Until December 31, 2018, Soc Trang had 1,204 patients on antiretroviral drugs (ARV), of which 88.2% used TDF-containing regimens and 98% of new patients received ARV in 2018 with TDF + 3TC + EFV regimen [6]. However, TDF induced nephrotoxicity cases have been reported [7], approximately 41% of patients taking TDF based regimen after 10 years treatment, which resulted in both acute kidney injury and chronic kidney disease occurring during treatment; cumulative events increased with duration of treatment [8]. According to Decision 5418/QD-BYT of the Ministry of Health in 2017 stipulating monitoring serum creatinine test before and during ARV treatment, this test was performed every 6–12 months or when renal dysfunction was suspected [3, 4]. Many studies showed that old age, low BMI, gender, CD4 less than 200 cells/mm^3^, long duration of HIV infection, WHO stage, and coinfection with hepatitis B and hepatitis C were risk factors for renal dysfunction [8–17]. A study has only followed up HIV patients treated with TDF based regimen within a duration of 24 months [9]. Little is known about the prevalence of renal impairment and risk factors among HIV-infected patients treated with TDF based regimen greater than 3 years. Our descriptive cross-sectional study aimed to estimate the prevalence and determine the correlation of renal impairment among this population.

## 2. Materials and Methods

This is a cross-sectional study conducted in the department of infectious diseases, general hospital, Soc Trang Province from November 2018 to March 2019. The study protocol was reviewed and approved by the review board of the general hospital, Soc Trang Province on October 25, 2018. All participants provided written informed consent forms.

### 2.1. Study Population and Study Design

**Figure 1:**
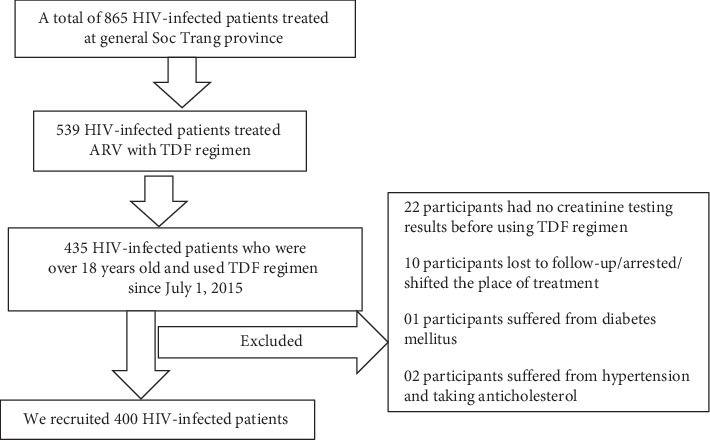
Enrollment of participants in Soc Trang, Vietnam.

The target population of this study was HIV-infected patients, excluding pregnant women, who voluntarily participated in this study, aged over 18 years, had eGFR greater than 60 ml/min/1.73 m^2^, and were treatment-naive or switched TDF regimen within over 3 years. The study was powered to obtain 80% power with a two-sided 5% significant level for estimating the overall prevalence of 50% with the desired precision of 5%, which required 384 HIV-infected patients; this was rounded up 400 to allow approximately 5% participant refusal and specimen damage rate (Figure 1).

This study followed two-stage recruitment strategy. First, based on the list of patients who received antiretroviral treatment at study setting (estimated 865 participants), we filtered all patients using TDF regimen over 36 months since July first, 2018 (approximately 435 participants). Second, we recruited participants admitted to the hospital for reexamination to collect data and take blood samples based on the treatment-naive regime until we recruited enough 400 participants.

### 2.2. Demographic Collection

The information sheet describing the purpose and method was shown to participants, and after giving informed consent, we collected baseline demographic and clinical data from medical records including sex, age, weight, height, HIV transmission, WHO stage, tested time for HIV positive, treatment time, cotrimoxazole, isoniazid, viral load, baseline CD4 count, baseline serum creatinine, ALT, AST, hepatitis B, hepatitis C, WHO stage in which treatment started, and baseline hemoglobin. Neither names nor detecting information like phone numbers or addresses were collected.

### 2.3. Specimen Collection

After the completed physical examination, 03 mL blood specimens were collected and stored at −20°C and transported to the Center of Testing Calibration, University of Medicine and Pharmacy at Ho Chi Minh City for serum creatinine, ALT, AST analysis by chemistry analyzer (AU480, Beckman Coulter, USA). Hemoglobin (Unicel DxH 800, Beckman Coulter, USA), CD4 cell count (cell/mm^3^, measured by the FacsCount, Becton Dickinson), and viral load (COBAS® AmpliPrep/COBAS® TaqMan® HIV-1 Test) testing were performed at general hospital Soc Trang, Center of HIV/AIDS prevention, Can Tho Province, and Pasteur Institute Ho Chi Minh City, Vietnam, respectively, between October 2018 and March 2019.

Measurement of weight and height: we used Health-scale weight scales and height ruler (made in Shanghai, China) to measure weight and height. Body mass index (BMI) was calculated as BMI = weight (in kg) ÷ (height (in m))^2^. According to the guideline of CKD EPI equation [18] to calculate the estimated glomerular filtration rate (eGFR) in HIV infected patients, serum creatinine values are used to calculate eGFR in the following equations below:

- For female with serum creatinine ≤0.7 mg/dl: GFR =166 × (Scr (in mg/dl)/0.7)^−0.329^ × (0.993)^Age (in year)^; female with serum creatinine >0.7 mg/dl: GFR = 166× (Scr (in mg/dl)/0.7)^−1.209^ × (0.993)^Age (in year)^
- For male with serum creatinine ≤0.9 mg/dl: GFR =163 × (Scr (in mg/dl)/0.9)^−0.411^ × (0.993)^Age (in year)^; female with serum creatinine >0.9 mg/dl: GFR = 163× (Scr (in mg/dl)/0.9)^−1.209^ × (0.993)^Age (in year)^

In this study, we used the Kidney Disease Outcomes Quality Initiative (KDOQI) of the National Kidney Foundation to clarify the eGFR (ml/min/1.73 m^2^) group including 6 stages (stage 1 (>90), stage 2 (60–89), stage 3a (45–59), stage 3b (30–44), stage 4 (15–29), and stage 5 (<15)).

### 2.4. Statistical Analysis

All information sheets were checked for missing data and were stored in locked cabinets. Data were entered using Epi-Data version 3.1 (Epi-Data Association, Odense, Denmark), and all statistical analysis was implemented using Stata version 13.0 (StataCorp, TX), and *P*-value < 0.05 was considered statistically significant.

The patient's characteristics were described using frequency and proportion for categorical variables and mean and standard deviation or median and interquartile ranges for continuous variables.

In this study, renal impairment was defined as eGFR less than 60 ml/min/1.73 m^2^ [13]. In the univariate analysis, we used Chi-squared and Fisher's exact tests to assess bivariate relationships. We used the prevalence ratio (PR) as a reliable and interpretable measure to predict renal impairment in the multivariate regression model. Variables that yielded a *P* less than 0.25 or were previously known to be an important risk factor in existing literature were included in multivariate analysis. In multivariate analysis, we used Poisson multivariate regression with backward elimination to detect the final model that describes contributing variables.

## 3. Results

### 3.1. Demographic Characteristics

A total of 400 participants were recruited in the present study. The mean (±SD) age was 39.8 (±8.8); 51.5% of respondents were male. The mean (±SD) age was 21.2 (±3.4).

### 3.2. Clinical Characteristics

The majority of study participants suffered from HIV through sexuality (89.5%); up to 98.3% of respondents were found in the World Health Organization (WHO) stage 1, and fifty percent recorded WHO stage 3 for starting treatment.

The mean (±SD) time for HIV positive testing and treatment was 7.0 (±7.0); 6.5 (±2.5), respectively. Up to 93.5% and 94.7% of respondents were preexposed to isoniazid and cotrimoxazole preventive therapy. The majority of study participants had a viral load less than 20 copies per mL (93.5%), and the mean (±SD) CD4 count, serum creatinine *µ*mol/L, and hemoglobin (g/L) were 517 (±250), 75.7 (±18.2), and 13.5 (±1.6), respectively.

9.7% of study participants were with comorbidity of hepatitis B and hepatitis C (7.2%). Among study participants, approximately one-third had an AST > 37 U/L and ALT > 40 U/L, and 26.2% had an AST > 37 U/L and ALT > 40 U/L (Table 1).

### 3.3. Renal Function Characteristics

In the present study, the mean (±SD) eGFR (ml/min/1.73 m^2^) was 96.0 (±24.5), 57.3% of respondents had baseline eGFR greater than 90 ml/min/1.73 m^2^, and none of them had had baseline eGFR less than 15 ml/min/1.73 m^2^, out of 34 (8.5%) of participants who had renal impairments (Table 1).

### 3.4. Subclinical Characteristics and KDIGO 2012

There were significant differences between the mean (±SD) baseline creatinine, eGFR, baseline CD4 count, and KDIGO stage among study participants (*P* < 0.001). Compared with KDIGO stage 1, patients who were diagnosed with KDIGO stages 2, 3, and 4 had significantly higher mean (±SD) baseline creatinine (70.1 (±12.6) vs 77.5 (±13.7), 97.2 (±18.8), 151.6 (±51.5), 167.9 (±16.9)), respectively. By contrast, compared with KDIGO stage 1, those with KDIGO stages 2, 3, and 4 had significantly lower mean (±SD) baseline eGFR, (111.9 (±18.1) vs 80.3 (±6.6), 56.4 (±3.7), 40.7 (±3.9), 21.8 (±5.5)), and mean (±SD) CD4 count (531.5 (±233.8) vs 530.7 (±404.9), 470.2 (±251.2), 526.5 (±345.8), 130.7 (±24.4)), respectively (Table 2).

### 3.5. Factors between Renal Impairment and Demographic and Clinical Characteristics among HIV/AIDS Patients

In univariate analysis, renal impairment was more prevalent among older HIV/AIDS patients; those had higher WHO stage and baseline CD4 count less than 200 cells/mm^3^. The proportion of renal impairment was less prevalent among those who were preexposed to isoniazid, with BMI more than 18.5 kg/m^2^.

However, after they were adjusted with a multivariate regression model, only three factors were associated with renal impairment in the final model, including age group, preexposure to isoniazid, and BMI group. Patients who aged more than 60 years (adjusted PR [aPR] = 26.75, 95% Cl: 3.38–211.62) compared with those aged 20–29 years had significantly increased risk of renal impairment. Patients who had BMI ranging from 18.5 to 25 kg/m^2^ (aPR = 0.31 Cl: 0.15–0.62) compared to BMI less than 18.5 kg/m^2^ and who were preexposed to isoniazid (aPR = 0.35 Cl: 0.14–0.91) were less likely to suffer from renal impairment (Table 3).

## 4. Discussion

In this study, we present findings from the renal impairment prevalence survey of semiurban HIV/AIDS patients conducted in southern Vietnam. A striking finding in this study is that participants who were preexposed to isoniazid and TDF were less likely to report renal impairment compared with those who were not preexposed to isoniazid. According to Decision No. 2495/QD-BYT dated July 18, 2012 [19], on issuing guidelines for active detection of tuberculosis and isoniazid prophylaxis in HIV infected patients; Decision No. 5418/QD-BYT dated December 1, 2017, on issuing HIV/AIDs treatment and care [3]; Decision No. 471/QD-BYT dated February 11, 2014, on issuing improving care and treatment quality among HIV infected patients of the Ministry of Health, Vietnam, HIV infected patients were screened for tuberculosis on each visit. In this study, 40 (10%) of the participants were coinfected with tuberculosis in which 379 (94.7%) had a cough, fever, night sweats, and weight loss; all of them were provided isoniazid prophylaxis for 9 months. Although some studies reported the influence of renal impairment on isoniazid use among tuberculosis patients [20, 21], a scarcity of evidence showed that isoniazid use affected renal function among HIV/AIDS treated with TDF regimen. Thus, the impact of preexposure to isoniazid and TDF should be carefully considered in patients for long-time monitoring of renal function.

We also found a low prevalence of renal impairment among study participants. This finding was consistent with findings in South Africa (7.6%) [13] and Malaysia 9.3% [17], lower than in Korean (14.4%) [22] and Japan (22.1%) [12], and higher than in Spain (4-5%) [23]. This could be explained that differences in genetic factors correlated with an inherited polymorphism in drug transporter proteins of the renal tubule which led to TDF accumulation in the proximal tubular cells [24, 25], and several studies also showed that HIV affected direct renal pathogenic role [13, 26, 27].

In the current study, we also found that patients who had low BMI were significantly associated with renal impairment compared with those who had normal BMI; this result was similar to studies conducted in Vietnam and Japan [9, 28, 29], but in contrast to the study conducted in Ethiopia [25]. It may be different sample sizes [25], duration of study time, as a little is known about BMI effect on renal function on the long term [9]; therefore, the need for deep and long-term research should be taken into account.

As shown in our study, we did not find the relationship between cotrimoxazole, hepatitis B, C virus infection, and renal impairment which was consistent with the study conducted in Vietnam [9]; however, these predictors could be affected by renal impairment [30, 31]. There may have been a difference in exclusion criteria in these studies due to the study design; therefore, participants affected by these risk factors could be excepted [9].

Compared with patients aged 20–29 years, patients with ages equal to and greater than 60 years were more likely to report renal impairment. This finding was in agreement with studies conducted in Ethiopia [25] and Malaysia [10], which showed that old age was considered to be a risk factor for TDF induced nephrotoxicity. It is known that kidney function and structure would deteriorate by age [25, 32].

In the univariate analysis, we found that baseline CD4 count less than 200 cell/mm^3^ was significantly associated with renal impairment, which was in line with the previous studies [25, 33, 34]. But in multivariate analysis, baseline CD4 count was not a risk factor; this may be partly explained by the fact that participants in the present study were receiving stable HIV treatment. However, lower baseline CD4 count was a predominant factor associated with kidney injury [25, 35].

The current study was the first study to assess the risk factors among participants who were treated with ARV based TDF regimen over 3 years in Vietnam. However, there are several limitations. In this present study, we did not collect urine data for analysis so that we could not assess chronic kidney disease among participants [36]. In a rural resource-limited setting of Soc Trang, Vietnam, proteinuria was not routinely measured, although proteinuria is frequently detected in HIV infected patients, with approximately thirty percent showing microalbuminuria despite normal serum creatinine levels [37]. In HIV infected patients, proteinuria is more likely to be progressing to AIDS and death. It could be explained that HIV-associated nephropathy demonstrates as rapidly progressive renal disease, associated with the severity of proteinuria levels and direct HIV infection of renal tissue [38]. Therefore the lack of infrastructure for such regular monitoring can make early detection of renal toxicity among HIV infected patients difficult to follow [6]. We recommended that routine proteinuria screening of all new patients should be taken into account at primary health care. Furthermore, due to cross-sectional design, we could only find the risk factors associated with renal impairment, but could not determine the causative association between TDF and renal function [29].

This survey has important implications for the prioritized strategies for the prevention of renal impairment as TDF is still an important drug for HIV-infected patients [9]. This study indicated low BMI, old age, and no exposure to isoniazid among HIV-infected patients treated with ARV based on TDF, which are primary risk factors for renal impairment. We strongly recommend the need for longitudinal studies to assess the influence of TDF on renal impairment.

## 5. Conclusion

Our findings underscore the need for longitudinal studies to assess the influence of TDF to maintain the low prevalence of renal impairment among HIV-infected patients in Vietnam.

## Figures and Tables

**Table 1:** Demographic, clinical characteristic among HIV-infected patients in Soc Trang, Vietnam.

Characteristics	Frequency (*n* = 400)	Percentage (%)
Sex		
Male	206	51.5
Female	194	48.5
Age (in year)	39.8 ± 8.8	Min: 20–max: 69
Age group		
20–29	38	9.5
30–39	181	45.2
40–49	127	31.8
50–59	43	10.8
≥ 60	11	2.7
BMI (in kg/m^2^)	21.2 ± 3.4	Min: 12.9–max: 35.0
BMI group		
<18.5	85	21.2
18.5–25	268	67.0
>25	47	11.8
HIV transmission		
Drug addiction	30	7.5
Sexuality	358	89.5
Mother to child	12	3.00
WHO stage (current)		
Stage 1	393	98.3
Stage 2	3	0.7
Stage 3	2	0.5
Stage 4	2	0.5
Interval of HIV infection after diagnosis (years)	7.0 ± 7.0	Min: 4–max: 20
Interval of HIV infection after diagnosis group (year)	7.0 ± 7.0	Min: 4–max: 20
<5	87	21.8
5–10	266	66.5
11–15	44	11.0
>15	3	0.7
Treatment time (year)	6.5 ± 2.5	Min: 3–max: 18
3–5	168	42.0
6–10	199	49.7
>10	33	8.3
Preexposure to cotrimoxazole		
Yes	374	93.5
No	26	6.5
Preexposure to isoniazid		
Yes	379	94.7
No	21	5.3
Viral load (<20 copies/ml)		
Yes	374	93.5
No	26	6.5
Baseline CD4 count (cells/mm^3^)	517.0 ± 250	Min: 37–max: 1691
>500 CD4 count	193	48.3
350–499 CD4 count	102	25.5
200–349 CD4 count	82	20.5
<200 CD4 count	23	5.7
Baseline serum creatinine (*µ*mol/L)	75.7 ± 18.2	Min: 45.7–max: 188
Baseline eGFR (ml/min/1.73 m^2^)	96.0 ± 24.5	Min: 15.8–max: 177.6
Stage 1 (>90)	229	57.3
Stage 2 (60–89)	137	34.2
Stage 3a (45–59)	29	7.2
Stage 3b (30–44)	2	0.5
Stage 4 (15–29)	3	0.8
Stage 5 (<15)	0	0.0
AST (>37 U/L)		
Yes	138	34.5
No	262	65.5
ALT (>40 U/L)		
Yes	140	35.0
No	260	65.0
AST (>37 U/L) and ALT (>40 U/L)		
Yes	105	26.2
No	285	73.8
Hepatitis B		
Yes	39	9.7
No	361	91.3
Hepatitis C		
Yes	29	7.2
No	371	92.8
WHO stage (started treatment)		
Stage 1	69	17.3
Stage 2	44	11.0
Stage 3	200	50.0
Stage 4	87	21.7
Baseline hemoglobin (g/L)	13.5 ± 1.6	Min: 6.8–max: 17.1
eGFR categories (mL/min/1.73 m^2^)		
Stage 1 (>90)	229	57.2
Stage 2 (60–89)	137	34.2
Stage 3a (45–59)	29	7.3
Stage 3b (30–44)	2	0.5
Stage 4 (15–29)	3	0.8
Stage 5 (<15)	0	0.0

**Table 2:** Subclinical stages according to Kidney Disease Improving Global Outcomes (KDIGO) 2012 among HIV-infected patients in Soc Trang, Vietnam.

Variables	KDIGO 2012					*P*
	Stage 1 *n* = 229	Stage 2 *n* = 137	Stage 3a *n* = 29	Stage 3b *n* = 2	Stage 4 *n* = 3	
Creatinine	70.1 ± 12.6	77.5 ± 13.7	97.2 ± 18.8	151.6 ± 51.5	167.9 ± 16.9	<0.01
eGFR	111.9 ± 18.1	80.3 ± 6.6	56.4 ± 3.7	40.7 ± 3.9	21.8 ± 5.5	<0.01
AST	41.6 ± 37.8	38.4 ± 27.2	34.4 ± 12.4	53.4 ± 12.8	71.6 ± 59.5	0.16
ALT	44.4 ± 42.2	37.9 ± 28.1	31.8 ± 17.3	74.2 ± 69.4	62.9 ± 33.1	0.14
Hb	13.6 ± 1.7	13.4 ± 1.6	13.4 ± 1.3	14.3 ± 0.7	12.5 ± 0.7	0.25
CD4 count	531.5 ± 233.8	530.7 ± 404.9	470.2 ± 251.2	526.5 ± 345.8	130.7 ± 24.4	0.02

**Table 3:** Predictors for renal impairment (KDIGO 2012) among HIV-infected patients in Soc Trang, Vietnam, with univariate and multivariate models.

Variables	Renal impairment % for each category				Univariate analysis	Multivariate analysis
	*N*	*N*	%	*P*	PR (95% CI)	aPR (95% CI)
Sex						
Female	194	16	8.3	0.860	Ref	
Male	206	18	8.7		1.06 (0.54–2.08)	
Age group						
20–29	38	1	2.6	<0.001	Ref	Ref
30–39	181	7	3.9		1.47 (0.18–11.94)	1.35 (0.16–11.01)
40–49	127	6	4.7		1.79 (0.22–14.91)	2.06 (0.14–17.14)
50–59	43	11	25.6		9.72 (1.25–75.29)^¥^	7.72 (0.99–60.14)
≥60	11	9	81.8		31.09 (3.94–245.40)^§^	26.75 (3.38–211.62)^¥^
HIV transmission						
Drug addition	30	2	6.7	0.510	Ref	
Sexuality	358	32	8.9		1.34 (0.32–5.59)	
Mother to child	12	0	0.0		—	
BMI						
<18.5	85	18	21.2	<0.001	Ref	Ref
18.5–25	268	15	5.6		0.26 (0.13–0.75)^¥^	0.31 (0.15–0.62)^¥^
>25	47	1	2.1		0.13 (1.33–0.52)^¥^	0.19 (0.02–1.48)
WHO stage (current)						
Stage 1	393	29	7.4	<0.001	Ref	Ref
Stage 2	3	2	66.7		9.03 (2.15–37.86)^§^	1.88 (0.24–14.94)
Stage 3	2	2	100.0		13.55 (3.23–56.79)^§^	1.67 (0.20–14.12)
Stage 4	2	1	50.0		6.77 (0.92–49.74)	2.40 (0.24–24.09)
Tested time for HIV positive (years)						
<5	87	9	10.3	0.840	Ref	
5–10	266	21	7.9		1.31 (0.60–2.86)	
11–15	44	4	9.1		1.15 (0.40–3.35)	
>15	3	0	0.0		—	
Treatment time						
3–5	168	14	8.3	0.980	Ref	
6–10	199	17	8.5		1.02 (0.50–2.08)	
>10	33	3	9.1		1.09 (0.31–3.79)	
Preexposure to cotrimoxazole						
No	26	3	11.5	0.560	Ref	
Yes	374	31	8.3		0.72 (0.22–2.34)	
Preexposure to isoniazid						
No	21	5	23.8	0.010	Ref	Ref
Yes	379	29	7.7		0.32 (0.12–0.83)^¥^	0.35 (0.14–0.91)^¥^
Viral load (<20 copies/ml)						
Yes	374	31	8.3	0.560	Ref	
No	26	3	11.5		0.72 (0.22–2.35)	
Baseline CD4 count (cells/mm^3^)						
>500 CD4 count	193	15	7.8	0.002	Ref	Ref
350–499 CD4 count	102	3	2.9		0.38 (0.11–1.31)	1.93 (0.43–8.73)
200–349 CD4 count	82	10	12.2		1.57 (0.70–3.49)	0.61 (0.10–3.71)
<200 CD4 count	23	6	26.1		3.35 (1.30–8.65)^¥^	1.20 (0.28–5.20)
AST (>37 U/L)						
No	262	19	7.3	0.210	Ref	
Yes	138	15	10.9		1.50 (0.76–2.94)	
ALT (>40 U/L)						
No	260	25	9.6	0.270	Ref	
Yes	140	9	6.4		0.67 (0.31–1.43)	
AST (>37 U/L) and ALT (>40 U/L)						
No	295	27	9.2	0.430	Ref	
Yes	105	7	6.7		0.73 (0.31–1.67)	
Hemoglobin (>16 g/L)						
No	377	33	8.8	0.460	Ref	
Yes	23	1	4.4		0.49 (0.68–3.36)	
Hemoglobin (<12 g/L)						
No	340	29	8.5	0.960	Ref	
Yes	60	5	8.3		0.97 (0.38–2.52)	
WHO stage (started treatment)						
Stage 1	69	1	1.5	0.130	Ref	
Stage 2	44	5	11.4		7.84 (0.92–67.11)	
Stage 3	200	19	9.5		6.55 (0.87–48.96)	
Stage 4	87	9	10.3		7.14 (0.90–56.34)	
Hepatitis B						
No	361	32	8.9	0.427	Ref	
Yes	39	2	5.1		1.58 (0.14–2.41)	
Hepatitis C						
No	371	32	8.6	0.750	Ref	
Yes	29	2	6.9		0.80 (0.19–3.33)	

^¥^<0.05. ^§^<0.001. aPR: adjusted prevalence ratio.

## Data Availability

The data used to support the findings of this study are available from the corresponding author upon request.

## Abbreviations

BMI: Body mass index
CD4: cluster of differentiation 4
eGFR: estimated glomerular filtration rate
HIV/AIDS: Human immunodeficiency virus infection and acquired immune deficiency syndrome
KDIGO: Kidney Disease Improving Global Outcomes
SD: Standard deviation
TDF: Tenofovir Disoproxil Fumarate
WHO: World Health Organization

## References

[1] Dispatch No. 796/BC-BYT, the Report for Prevention and HIV/AIDS in the First Six Months and Key Mission in the Last Six Months, (Suppl_A), August 09, 2016

[2] Mouton J. P., Cohen K., Maartens G. (2016). Key toxicity issues with the WHO-recommended first-line antiretroviral therapy regimen. *Expert Review of Clinical Pharmacology*.

[3] Ministry of Health, Decision No. 5418/QD-BYT Dated December 1, 2017, of the Ministry of Health Promulgating the Guidelines for HIV/AIDS Treatment and Care, 2017, http://vaac.gov.vn/vanban_detail/Detail/Quyet-dinh-so-5418-QD-BYT-ngay-01-12-2017-cua-Bo-Y-te-ve-viec-ban-hanh-Huong-dan-dieu-tri-va-cham-soc-HIV-AIDS

[4] Ministry of Health, Decision No. 3047/QD-BYT Dated July 22, 2015, of the Ministry of Health Promulgating the Guidelines for HIV/AIDS Treatment and Care, 2015, http://vaac.gov.vn/vanban_detail/Detail/?userkey=Quyet-dinh-3047-2015-QD-BYT-ve-viec-ban-hanh-Huong-dan-quan-ly-dieu-tri-va-cham-soc-HIV-AIDS

[5] Kumarasamy N., Sundaram S., Poongulali S., Ezhilarasi C., Pradeep A., Chitra D. (2018). Prevalence and factors associated with renal dysfunction in patients on tenofovir disoproxil fumarate-based antiretroviral regimens for HIV infection in Southern India. *Journal of Virus Eradication*.

[6] Center of HIV/AIDS Prevention in Soc Trang Province (2019). *Summary Report on HIV/AIDS Prevention and Control in Soc Trang Province 2018*.

[7] Waheed S., Attia D., Estrella M. M. (2015). Proximal tubular dysfunction and kidney injury associated with tenofovir in HIV patients: a case series. *Clinical Kidney Journal*.

[8] Nishijima T., Kawasaki Y., Tanaka N. (2014). Long-term exposure to tenofovir continuously decrease renal function in HIV-1-infected patients with low body weight. *AIDS*.

[9] Mizushima D., Tanuma J., Dung N. T. (2014). Low body weight and tenofovir use are risk factors for renal dysfunction in Vietnamese HIV-infected patients. A prospective 18-month observation study. *Journal of Infection and Chemotherapy*.

[10] Kumar S., Koh H. M. (2016). Tenofovir-induced nephrotoxicity: a retrospective cohort study. *Medical Journal*.

[11] Pujari Sanjay N., Smith C., Abhimanyu M. (2014). Higher risk of renal impairment associated with tenofovir use amongst people living with HIV in India: a comparative cohort analysis between Western India and United Kingdom. *BMC Infectious Diseases*.

[12] Suzuki S., Nishijima T., Kawasaki Y. (2017). Effect of tenofovir disoproxil fumarate on incidence of chronic kidney disease and rate of estimated glomerular filtration rate decrement in HIV-1-Infected treatment-naïve Asian patients: results from 12-year observational cohort. *AIDS Patient Care and STDs*.

[13] Assaram S., Mashamba-Thompson T. P., và Magula N. P. (2018). Risk factors and co-morbidities associated with changes in renal function among antiretroviral treatment-naive adults in South Africa: a chart review. *Southern African Journal of HIV Medicine*.

[14] Woolnough E. L., Hoy J. F., Cheng A. C. (2018). Predictors of chronic kidney disease and utility of risk prediction scores in HIV positive individuals. *AIDS*.

[15] Nagalingeswaran K. (2018). Prevalence and factors associated with renal dysfunction inpatients on tenofovir disoproxil fumarate-based antiretroviralregimens for HIV infection in Southern India. *Journal of Virus Eradication*.

[16] Monika K. (2015). Renal impairment in HIV-infected patients initiating tenofovir-containing antiretroviral therapy regimens in a primary healthcare setting in South Africa. *Tropical Medicine & International Health*.

[17] Koh H. M., và Suresh K. (2016). Tenofovir-induced nephrotoxicity: a retrospective cohort study. *Medical Journal of Malaysia*.

[18] Levey A. S., Stevens L. A., Schmid C. H. (2009). A new equation to estimate glomerular filtration rate. *Annals of Internal Medicine*.

[19] Ministry of Health *Decision No. 2495/QD-BYT Dated July 18, 2012, of the Ministry of Health on Issuing Guidelines for Active Detection of Tuberculosis and Isoniazid Prophylaxis in HIV-Infected Patients*.

[20] Bowersox D. W., Winterbauer R. H., Stewart G. L., Orme B., Barron E. (1973). Isoniazid dosage in patients with renal failure. *New England Journal of Medicine*.

[21] Temprano ANRS 12136 Study Group (2015). A trial of early antiretrovirals and isoniazid preventive therapy in Africa. *New England Journal of Medicine*.

[22] Lee J. E., Lee S., Song S. H. (2017). Incidence and risk factors for tenofovir-associated nephrotoxicity among human immunodeficiency virus-infected patients in Korea. *The Korean Journal of Internal Medicine*.

[23] Juega-Marino J., Bonjoch A., Perez-Alvarez N. (2017). Prevalence, evolution, and related risk factors of kidney disease among Spanish HIV-infected individuals. *Medicine (Baltimore)*.

[24] Jafari A., Khalili H., Dashti-Khavidaki S. (2014). Tenofovir-induced nephrotoxicity: incidence, mechanism, risk factors, prognosis and proposed agents for prevention. *European Journal of Clinical Pharmacology*.

[25] Yazie T. S., Orjino T. A., Degu W. A. (2019). Reduced kidney function in tenofovir disoproxil fumarate based regimen and associated factors: a hospital based prospective observational study in Ethiopian patients. *International Journal of Nephrology*.

[26] Krawczyk C. S., Holmberg S. D., Moorman A. C., Gardner L. I., McGwin G. (2004). Factors associated with chronic renal failure in HIV-infected ambulatory patients. *AIDS*.

[27] Ross M. J., Klotman P. E. (2002). Recent progress in HIV-associated nephropathy. *Journal of the American Society of Nephrology*.

[28] Nishijima T., Komatsu H., Gatanaga H. (2011). Impact of small body weight on tenofovir-associated renal dysfunction in HIV infected patients: a retrospective cohort study of Japanese patients. *PLoS One*.

[29] Mizushima D., Tanuma J., Kanaya F. (2013). WHO antiretroviral therapy guidelines 2010 and impact of Tenofovir on chronic kidney disease in Vietnamese HIV-infected patients. *PLoS One*.

[30] Peters L., Grint D., Lundgren J. D. (2012). Hepatitis C virus viremia increases the incidence of chronic kidney disease in HIV infected patients. *AIDS*.

[31] Ryom L., Mocroft A., Kirk O. (2013). Association between antiretroviral exposure and renal impairment among HIV-positive persons with normal baseline renal function: the D:A:D study. *Journal of Infectious Diseases*.

[32] Kalayjian R. C., Lau B., Mechekano R. N. (2012). Risk factors for chronic kidney disease in a large cohort of HIV-1 infected individuals initiating antiretroviral therapy in routine care. *AIDS*.

[33] Choi A. I., Li Y., Parikh C., Volberding P. A., Shlipak M. G. (2010). Long-term clinical consequences of acute kidney injury in the HIV-infected. *Kidney International*.

[34] WHO (2016). *Consolidated Guidelines on the Use of Antiretroviral Drugs for Treating and Preventing HIV Infection: Recommendations for a Public Health Approach*.

[35] Gallant J. E., Moore R. D. (2009). Renal function with use of a tenofovir-containing initial antiretroviral regimen. *AIDS*.

[36] Chaisiri K., Bowonwatanuwong C., Kasettratat N., Kiertiburanakul S. (2010). Incidence and risk factors for tenofovir-associated renal function decline among Thai HIV-infected patients with low-body weight. *Current HIV Research*.

[37] Estrella M. M., Fine D. M. (2010). Screening for chronic kidney disease in HIV-infected patients. *Advances in Chronic Kidney Disease*.

[38] Venter W. D. F., Fabian J., Feldman C. (2018). An overview of tenofovir and renal disease for the HIV-treating clinician. *Southern African Journal of HIV Medicine*.

